# The neurochemistry and morphology of functionally identified corneal polymodal nociceptors and cold thermoreceptors

**DOI:** 10.1371/journal.pone.0195108

**Published:** 2018-03-28

**Authors:** Abdulhakeem S. Alamri, Rhiannon J. Wood, Jason J. Ivanusic, James A. Brock

**Affiliations:** Department of Anatomy and Neuroscience, The University of Melbourne, Melbourne, Victoria, Australia; University of Southern California, UNITED STATES

## Abstract

It is generally believed that the unencapsulated sensory nerve terminals of modality specific C- and Aδ-neurons lack structural specialization. Here we determined the morphology of functionally defined polymodal receptors and cold thermoreceptors in the guinea pig corneal epithelium. Polymodal receptors and cold thermoreceptors were identified by extracellular recording at the surface of the corneal epithelium. After marking the recording sites, corneas were processed to reveal immunoreactivity for the transient receptor potential channels TRPV1 (transient receptor potential cation channel, subfamily V, member 1) or TPRM8 (transient receptor potential cation channel subfamily M member 8). Polymodal receptor nerve terminals (n = 6) were TRPV1-immunoreactive and derived from an axon that ascended from the sub-basal plexus to the squamous cell layer where it branched into fibers that ran parallel to the corneal surface and terminated with small bulbar endings (ramifying endings). Cold thermoreceptor nerve terminals were TRPM8-immunoreactive (n = 6) and originated from an axon that branched as it ascended through the wing cell and squamous cell layers and terminated with large bulbar endings (complex endings). These findings indicate that modality specific corneal sensory neurons with unencapsulated nerve endings have distinct nerve terminal morphologies that are likely to relate to their function.

## Introduction

Combined functional and morphological studies have provided detailed information about the nerve terminal structure of the different types of sensory receptors formed by Aβ-fibers sensory neurons in tissues such as skin (e.g. see reviews by [1, 2]). In comparison, the morphology of functionally defined sensory receptors formed by the unencapsulated (free or naked) nerve endings of modality specific unmyelinated (C-fiber) and thinly myelinated (Aδ-fiber) sensory neurons remains largely unknown. Serial section electron microscopy has been used to reveal the fine 3-dimensional structure of functionally defined cold thermoreceptors in skin [3] and sclera [4]. Electron microscopy has also been used to reveal the fine structure of molecularly defined low threshold mechanoreceptors formed by C-fibers and Aδ-fibers associated with hair follicles [5, 6]. There are also studies using light microscopy in the intestine that have revealed the entire nerve terminal structure of functionally defined mechanoreceptors formed by extrinsic sensory neurons within enteric ganglia [7, 8]. While the free nerve endings formed by C- and Aδ-fiber sensory neurons are often described as lacking structural specialisation [9], these morphological studies indicate that different functional types of C- and Aδ-fiber neurons form distinct sensory nerve terminal structures.

The cornea has a relatively simple avascular epithelium with low metabolic demands and tissue transparency, and is very densely supplied by unmyelinated sensory axons that terminate with free nerve endings close to the surface of the epithelium [10–12]. These features make it an ideal tissue to study the structure and function of these nerve terminals. Using a small diameter (≤ 50 μm) suction electrode applied to the epithelial surface of the guinea pig cornea, nerve impulses originating in single sensory nerve terminals can be recorded [13]. Using locally applied stimuli, the nerve terminals giving rise to electrical activity at the surface of the guinea pig corneal epithelium can be identified as capsaicin-sensitive receptors that have low levels of ongoing activity (<1 impulse/s) or cold thermoreceptors that have a continuous rhythmic discharge of nerve impulses at normal corneal temperature (mean discharge frequency ~10 impulses/s) [13–15]. The capsaicin-sensitive receptors are also activated by mechanical stimuli and are therefore polymodal receptors [13].

In recent studies, we have used confocal microscopy to investigate sensory nerve terminations in the guinea pig corneal epithelium [16, 17]. For this analysis we only considered the nerve terminal structure of individual axons that branched from leash fibers in the sub-basal plexus (SBP) at the base of the epithelium. Polymodal receptors were defined by expression of the TRPV1 (transient receptor potential cation channel, subfamily V, member 1) channel, which is activated by noxious heat, protons and capsaicin [18]. Their axons either did not branch after leaving the leash fibers and terminated within the wing cell layer with a single bulbar ending (simple ending), or ascended to the squamous cell layer where they branched into three or four fibers that each ran parallel to the surface and terminated in a single bulbar ending (ramifying ending) [16]. In addition, there were a small number of TRPV1 expressing axons that terminated in the basal epithelium with simple endings. The TRPV1 expressing terminals could be further classified by expression of calcitonin gene-relate peptide (CGRP), which was only present in simple endings in the wing and basal cell layers [16, 17]. Cold thermoreceptors were defined by expression of the TRPM8 (transient receptor potential cation channel subfamily M member 8) channel, which is activated by cooling and is known to contribute to cold sensation [19]. Their axons branched as they ascended from the leash fibers through the wing cell layers and terminated with multiple, large bulbar endings in the wing and squamous cell layers (complex endings [17]). Based on these findings we hypothesized that there is morphological specialization of sensory nerve terminals within the corneal epithelium and that this might be related to the function of modality specific neurons.

The aim of this study was to identify the morphology and neurochemical profiles of functionally identified capsaicin-sensitive receptors with low levels of ongoing activity and cold thermoreceptors in the corneal epithelium of the guinea pig. To achieve this, sites on the surface of the corneal epithelium where focal extracellular recordings indicated the presence of one of these receptors were marked and the nerve terminals located beneath the electrode were investigated using immunohistochemistry to reveal both their immuno-reactivity to TRPV1 or the cold-transducer protein TRPM8, and their structure.

## Methods and materials

All experiments conformed to the Australian National Health and Medical Research Council code of practice for the use of animals in research, and were approved by the University of Melbourne Animal Experimentation Ethics Committee.

### Tissue preparation

Tricolored guinea pigs of both sexes and in the weight range 180–350 g were used. Animals were euthanized by stunning followed by severing of the carotid arteries. Both eyes were dissected free from their orbits and isolated along with a short length of optic nerve and associated ciliary nerves. During and following dissection the tissue was kept immersed in physiological saline containing: 133.4 mM NaCl; 4.7 mM KCl; 2 mM CaCl_2_; 1.2 mM MgCl_2_; 1.3 mM NaH_2_PO_3_; 16.3 mM NaHCO_3_; 7.8 mM glucose. This solution was gassed with 95% O_2_−5% CO_2_. After isolation the eyes were maintained in the physiological saline at room temperature for approximately 2 h before being mounted in the recording chamber. This holding period prevents rapid sloughing of the corneal epithelium upon re-warming to physiological temperature.

### Extracellular recording from nerve terminals

Eyes were mounted in a 5 ml recording chamber (see [13]) and continuously superfused at 5 ml min^−1^ with physiological saline. Under control conditions, the temperature of the bathing solution was maintained at ~32°C. The optic nerve and associated ciliary nerves were drawn into a suction-stimulating electrode. The ciliary nerves were stimulated electrically with a constant voltage stimulator (pulse width 0.1–0.5 ms, 5–30 V). To record electrical activity from sensory nerve terminals, a glass-recording electrode (tip diameter ~50 μm) filled with HEPES-buffered physiological saline (10 mM HEPES, 150.8 mM NaCl, 4.7 mM KCl, 2 mM CaCl2, 1.2 mM MgCl_2_; the pH of this solution was adjusted to 7.4 using NaOH) was applied to the surface of the corneal epithelium with slight suction. An Ag-AgCl electrode in the recording chamber served as the indifferent electrode. Electrical activity was amplified (1000×, Geneclamp 500, Axon Instruments), filtered (high pass 1 Hz, low pass 10 kHz; Neurolog, Digitimer Ltd, UK), digitized at 20 kHz (Powerlab, ADInstruments, Australia) and stored to disk using the recording software Chart (ADInstruments). Recordings were only made from sites on the corneal surface where the nerve terminal impulses (NTIs) were readily distinguished from the noise (~10 μV peak-to-peak). At many sites on the corneal surface, electrically evoked or ongoing electrical activity was either absent or too small to be analysed.

### Receptor identification

The data presented were collected at recording sites where the electrical activity originated from a single nerve terminal. At these sites, electrical stimulation of the ciliary nerves evoked a single stimulus-locked all-or-none NTI. Only NTIs that were defined as originating in either polymodal nociceptors or cold thermoreceptors were analysed. As previously described [13, 14], polymodal nociceptors were defined by their having no ongoing NTI discharge or a low level of spontaneous NTI discharge (<1 impulse/s) that occurred irregularly and by their rapid excitation when 0.5 μM capsaicin was added to the physiological saline superfusing the eye (within 30 seconds of switching the superfusing solutions). In the present experiments, the initiation of the response to capsaicin was defined by the point at which NTI frequency increased to >2 Hz; statistical comparison was made between the number of NTIs recorded during 20 s just prior to the initiation of the response and during the first 20 seconds of the response using a paired *t*-test. Brock et al. [13] reported that receptors with these properties were also activated by mechanical stimuli applied by advancing the recording electrode tip into the cornea. In the present study, mechanical stimuli were not applied because of the risk of displacing the recording electrode and/or damaging the nerve terminals at the surface of the epithelium. As previously described [15], cold thermoreceptors were defined by their relatively high levels of ongoing NTI discharge (typically around 10 impulses/s) that occurred in a rhythmic manner (i.e. occurred as single impulses or short bursts of impulses at relatively fixed intervals) and that was increased by cooling and decreased by warming the solution superfusing the cornea. For statistical comparison, we compared the frequency of NTI discharge during 5 s periods at the basal temperature (~32°C) and at the peak of the response during heating to ~40°C and cooling to ~28°C. Statistical comparisons were made with a repeated measures ANOVA followed by Bonferroni post hoc tests. All statistical tests were performed using IBM SPSS statistics for Windows (version 24, IBM Corp, Armonk, NY) and *P* values <0.05 were considered significant. All data are reported as mean and standard deviation (SD).

### Labeling the recording sites on the cornea

To allow the morphology of nerve terminals located at the recording site to be investigated, a polyethylene tube was positioned inside the recording electrode, close to the tip, to allow the solution in the recording electrode to be replaced with HEPES saline buffer containing 0.05% (w/v) fluoro-gold (Sigma-Aldrich, Australia). This was done after a recording site was identified and it was confirmed that only a single all-or-none NTI could be evoked by electrical stimulation. To ensure that the electrode position had not moved during the application of fluoro-gold, it was confirmed that electrical stimulation still evoked a single all-or-none NTI at a similar latency. After then confirming the modality of the receptor (see above), the electrode was removed and the eye placed in Zamboni’s fixative (2% (v/v) formalin and 15% (v/v) saturated picric acid in 0.1M phosphate buffer) at 4°C. After 10 min in fixative, the anterior segment together with ~1 mm of sclera was dissected from the eye and the lens and iris was carefully removed. The tissue was then fixed for 3 h in Zamboni’s fixative at 4°C.

### Immunohistochemistry

To identify the structure and neurochemical phenotype of the functionally identified polymodal nociceptors and cold thermoreceptors the fixed segment of the cornea containing the labeled recording site was washed 3 times with 0.1 M phosphate buffered saline (PBS). The tissue was then treated for 1 h with blocking solution containing 10% (v/v) normal horse serum prepared in antibody diluent (0.1M PBS containing 0.3% Triton X-100 and 0.1% sodium azide) and incubated overnight with rabbit anti-TRPV1 or rabbit anti-TRPM8 primary antibodies (see Table 1 for details of source and concentration). In all cases, the tissue was co-labeled with mouse anti-β-tubulin III (Table 1) to reveal all corneal axons. The following day, the tissue was washed 3 times with PBS and incubated for 2 h with secondary antibodies (see Table 1). All antibodies were diluted with antibody diluent. After further washing with PBS, the tissue was mounted and cover-slipped using DAKO fluorescence mounting medium (Carpinteria, CA).

**Table 1:** Source and concentrations of the primary and secondary antisera used in this study.

**Primary Antibody Antigen**	**Immunogen**	**Manufacturer**	**Dilution used**
TRPV1	C-terminus of rat TRPV1 (824–838)	Alomone labs, Jerusalem, Israel; rabbit polyclonal; # ACC030	1:1000
TRPM8	C-terminus (amino acids 1005–1104) of rat TRPM8	Supplied by Hiroshi Hosokawa; rabbit polyclonal [20]	1:5000
β-Tubulin III	Microtubules from rat brain	Covance, Emeryville, CA; mouse monoclonal; Cat # MMS-435P	1:2000
**Secondary antibody**	**Manufacturer**		**Dilution used**
Donkey α rabbit Alexa594	Molecular probes, Invitrogen; #A21207		1:200
Donkey α mouse Alexa488	Molecular probes, Invitrogen; #A11001		1:200

### Antibody characterisation

Anti-TRPV1 (Alomone Labs, ACC-030) is a rabbit polyclonal antibody raised against the intracellular c-terminus of rat TRPV1 (824–838). Western blotting of TRPV1 transfected HEK 293 cells shows a single band at 100 kDa (manufacturer’s technical information). Immuno-labeling of trigeminal neurons with this antibody is not present in TRPV1 knockout mice, but is present in wild-type controls [21]. Furthermore, we have performed experiments to show that pre-adsorption with the manufacturer’s peptide (TRPV1 peptide control antigen for Cat #ACC030, Lot #ACC030AG0440, Alomone labs) at 0.1–1 μg/ml completely abolishes staining in guinea pig corneal epithelium and that there was almost complete co-localization of the TRPV1 antibody staining and mRNA expression in guinea pig trigeminal neurons revealed by combining *in situ* hybridisation and immunohistochemistry [16].

Anti-TRPM8 antibody (supplied by Hiroshi Hosokawa) is a rabbit polyclonal antibody raised against the C-terminus (amino acids 1005–1104) of rat TRPM8 [20]. It produced cytoplasmic labeling of HEK293 cells transfected with pCMV-Myc-TRPM8 and a single band at ~130 kDa (close to the measured molecular weight of TRPM8) in Western blots of proteins isolated from these transfected cells [20]. No labeling was observed in mock transfected cells or when pre-absorbed with the antigen [20].

Mouse anti-β-tubulin III (MMS-435P Covance) is a monoclonal antibody (TUJ1 clone) that has been raised against microtubules derived from rat brain. It is specific to Class III β-tubulin and does not recognize β-tubulin found in glial cells (manufacturers’ information). This antibody recognizes an epitope located within the last 15 C-terminal residues of Class III β- tubulin and in Western blot analysis of rat brain this antibody identifies a single protein with the molecular weight (50 kDa) predicted for Class III β-tubulin [22,23]. In Western blots, this antibody has not shown any cross reaction with the isotype defining regions of chicken Class II or IV β-tubulin or human Class I, II, IV and V β-tubulin [24]. It does however cross react with the isotype defining region of both chicken and human Class III β-tubulin [24].

### Image collection

The tissues were imaged with x40 or x63 objectives on a Zeiss LSM510 Meta confocal microscope and collected using Zen imaging software (v5.5, Carl Zeiss MicroImaging, GmbH). The recording site was first identified by fluoro-gold fluorescence and its precise location was revealed by the mark left by the rim of the electrode on the corneal surface when viewed in bright-field. Three-dimensional reconstructions of the labeled nerve terminals were prepared using Imaris software (version 7.7.0, Bitplane AG, Zurich, Switzerland).

## Results

### Cold thermoreceptors

#### Functional properties of identified corneal cold thermoreceptors

**Fig 1:**
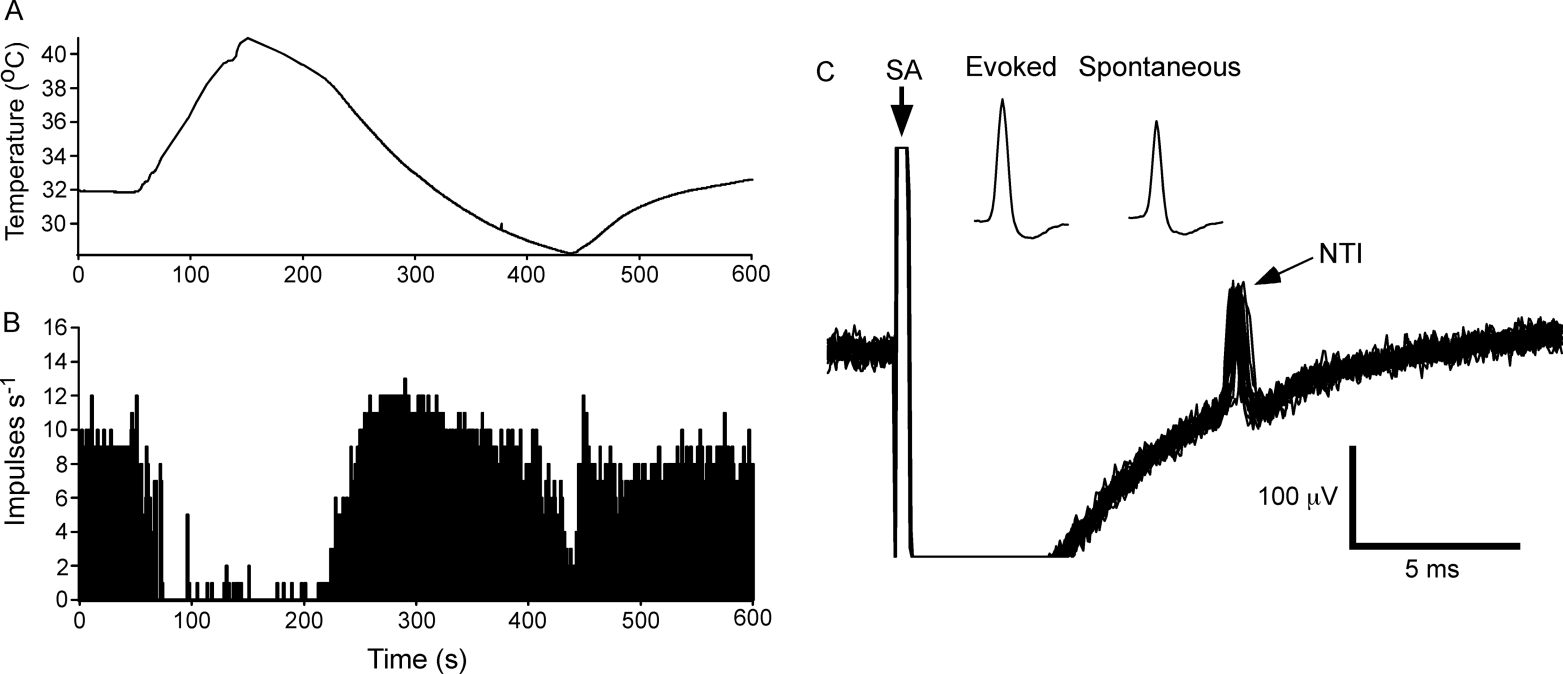
The electrical activity recorded from a cold thermoreceptor nerve terminal at the corneal surface. **A** and **B**, show the temperature of the solution superfusing the cornea (**A**) and the frequency of nerve terminal impulse (NTI) discharge (**B**). During heating and cooling the frequency of NTIs was decreased and increased, respectively. **C**, overlaid traces recorded during stimulation of the ciliary nerves with a train of 25 pulses at 1 Hz. At this recording site electrical stimulation evoked a single stimulus locked NTI. The insets in **C** show averages of the electrically evoked and spontaneous NTIs. The smaller amplitude of the spontaneous NTIs indicates they are likely to be initiated very close to site of recording (see [25]). In **C**, the stimulus artifact (SA) is indicated and during the flat line immediately following the SA the signal was out of the analogue-to-digital recording range.

Cold thermoreceptors (n = 12) were defined by the presence of a rhythmic ongoing discharge of NTIs (mean frequency 9.3 ± 2.8 impulses/s (Mean ± SD); the electrophysiological measurements and observational findings for each cold thermoreceptor are presented in S1 Table) that was increased by cooling (15.4 ± 3.0 impulses/s at the peak of the response to cooling, Bonferroni post hoc test *P* < 0.001) and markedly reduced or silenced by heating (0.6 ± 1.0 impulses/s at the peak of the response to warming, Bonferroni post hoc test *P* < 0.001; Fig 1A and 1B). At these sites, electrical stimulation of the ciliary nerves evoked a single stimulus-locked NTI (Fig 1C; average latency 8.0 ± 2.2 ms) and the spontaneously occurring NTIs could be collided with the electrically evoked NTIs preventing their arrival at the recording site (see [25]). These findings provide strong evidence that the recorded electrical activity arises from a single unit. As previously reported [25], the positive peak amplitude of the spontaneously occurring NTIs was smaller than that of electrically evoked NTIs (S1 Table and insets in Fig 1C).

#### Functionally defined corneal cold thermoreceptors express TRPM8 and have complex endings

**Fig 2:**
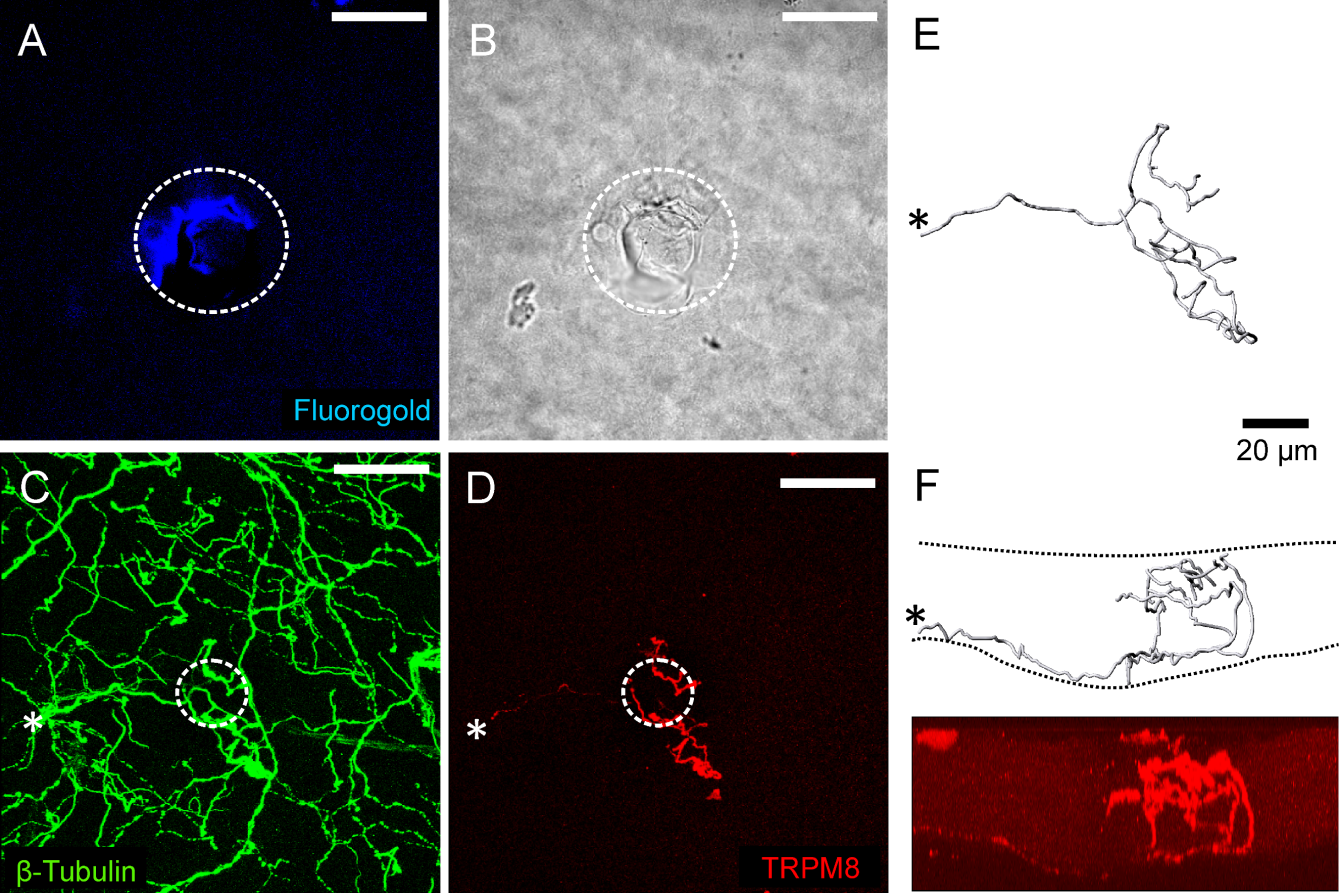
Immuno-labeling for TRPM8 at the cold thermoreceptor recording site where the electrical activity shown in Fig 1 was recorded. A, fluoro-gold fluorescence within the white dotted circle identifies the approximate location of the recording site. B, bright-field image showing within the white dotted circle the mark left on the corneal surface by the rim of the recording electrode. C, all axons in the field of view were revealed by β-tubulin III-IR (green). D, a TRPM8-IR (red) nerve terminal was located at the recording site. In C and D, the white dotted circle approximates dimensions of the electrode tip at the recording site revealed in B. The scale bar in A–D = 50 μm. E and F, show a 3-dimensional reconstruction of the TRPM8-IR nerve terminal viewed from the front (E) and side (F). Panel F shows an orthogonal projection of the image shown in D. In C–F, the asterisk marks the site where the parent axon of the TRPM8-IR nerve terminal enters into the epithelium through Bowman’s membrane. The dashed lines in F approximate the area occupied by the corneal epithelium.

Immuno-labeling for TRPM8 was assessed at 6 cold thermoreceptor recording sites and in all cases the axon terminals at the surface of the corneal epithelium immediately beneath the recording electrode were TRPM8-immunoreactive (-IR; for the receptor identified in Fig 1 see Fig 2). In these preparations, the cornea was also immuno-labeled for β-tubulin III (Fig 2C) and this demonstrated the presence of many axon terminals that were not TRPM8-IR in the area surrounding the recording site. There were no axon terminals that were only β-tubulin III-IR close to the surface of the cornea within the area enclosed by the recording electrode, confirming that only TRPM8-IR terminals produced the recorded electrical activity. In all cases, the axon branch(es) from the SBP that gave rise to the TRPM8-IR axon terminals under the recording electrode had complex morphology with irregular large *en passant* boutons, short (< 5 μm) axonal side branches in the wing cell and squamous cell layers, and multiple bulbar endings located close to the surface of the corneal epithelium (Fig 2E and 2F). Most of the axon branches from the SBP also had one or two major branch points as they ascended through wing cell layer and entered the squamous cell layer. In all cases, there was a cluster of 2 to 5 TRPM8 complex nerve terminal branches that terminated at the surface of the corneal epithelium in the immediate vicinity of the recording site. At 5 of the 6 recording sites, the axon branches forming the nerve terminal cluster clearly originated from the same parent axon in the SBP (e.g. see Fig 2E and 2F). These parent axons penetrated Bowman’s membrane (i.e. entered the SBP) close to the site of recording and formed only a single nerve terminal cluster in the corneal epithelium. In the remaining case, the axons in the SBP could not be followed unambiguously and it was therefore not possible to confirm that all the branches in the cluster under the recording electrode arose from the same parent axon.

#### Functionally defined corneal cold thermoreceptors terminals were not TRPV1-IR

**Fig 3:**
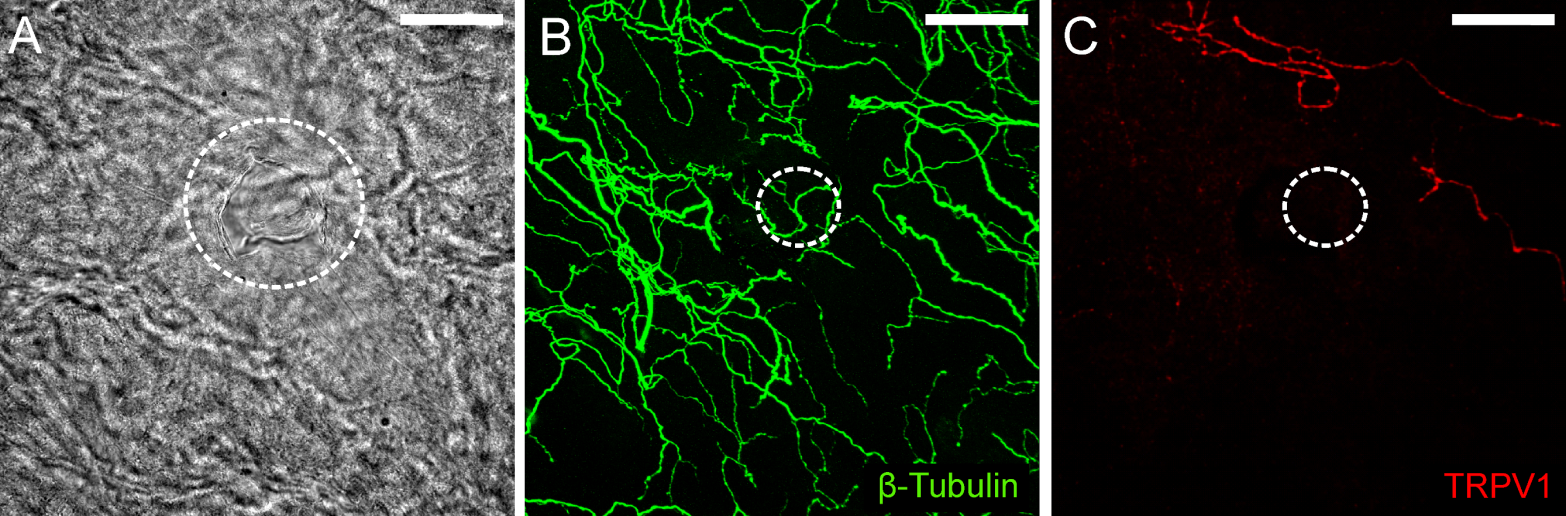
Immuno-labeling for TRPV1 at a cold thermoreceptor recording site. A, bright-field image showing within the white dotted circle the mark left on the corneal surface by the rim of the recording electrode. B, all axons in the field of view were revealed by β-tubulin III-IR (green). C, while TRPV1-IR axons (red) were observed within the field of view, those enclosed by the recording electrode were not immunoreactive for this protein. In B and C, the dotted circle approximates the dimensions of the electrode tip at the recording site revealed in A. The scale bar in A–C = 50 μm.

The possibility that cold thermoreceptors were also TRPV1-IR was investigated at the remaining 6 recording sites. At these recording sites, there were β-tubulin III-IR axon terminals located close to the surface of the corneal epithelium immediately beneath the recording electrode, but none of these were TRPV1-IR (Fig 3). In these experiments, it was confirmed that TRPV1-IR nerve terminals were present outside of the recording site (Fig 3C), so failure to demonstrate labeling of cold thermoreceptors for TRPV1 cannot be attributed to poor immuno-labeling.

### Polymodal nociceptors

#### Functional properties of identified corneal polymodal nociceptors

**Fig 4:**
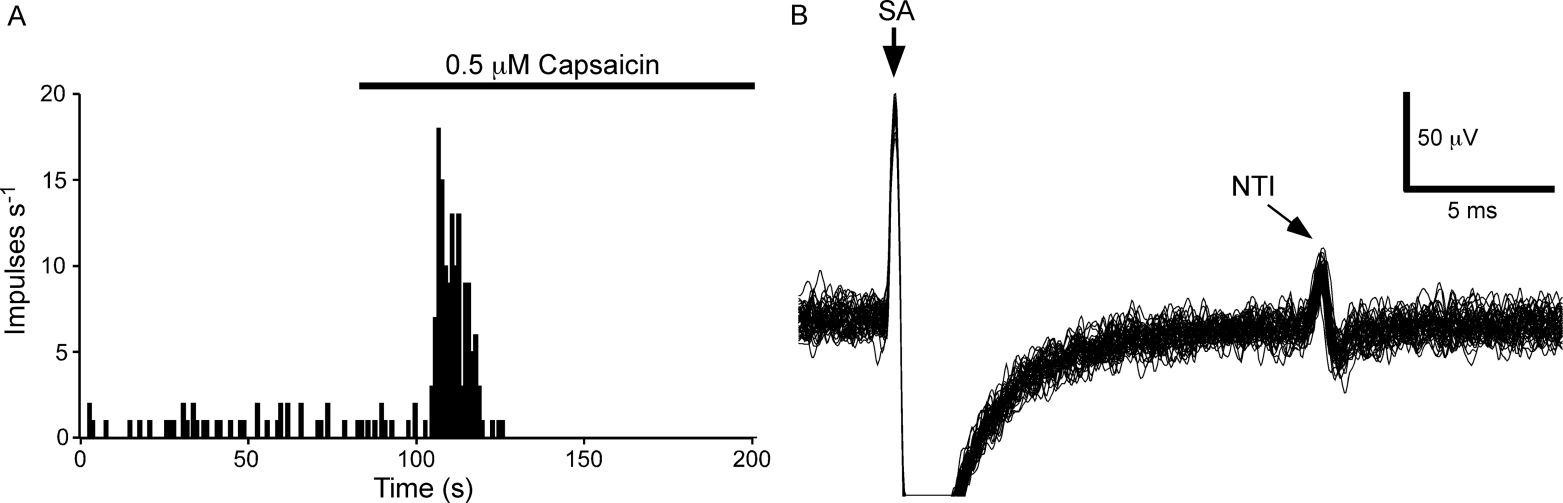
The electrical activity recorded from a capsaicin-sensitive nerve terminal at the corneal surface. A, the frequency of NTI discharge before and during application of capsaicin (0.5 μM). In this receptor, there was a low level of ongoing NTI activity that was markedly increased by capsaicin. B, overlaid traces recorded during stimulation of the ciliary nerves with a train of 25 pulses at 1 Hz. At this recording site electrical stimulation evoked a single stimulus locked NTI. In B, the stimulus artifact (SA) is indicated and during the flat line immediately following the SA the signal was out of the analogue-to-digital recording range.

Polymodal nociceptors (n = 9) were defined by their excitation by capasicin (Fig 4A). At these recording sites, electrical stimulation evoked a single stimulus-locked NTI (Fig 4B; average latency 11.4 ± 3.3 ms; the electrophysiological measurements and observational findings for each polymodal nociceptor are presented in S2 Table). At 5 of the 9 recording sites there was a low level of ongoing NTI discharge (average frequency 0.21 ± 0.26 impulses/s). In these recordings, there were 4.1 ± 5.1 spikes during the 20 s prior to excitation with capsaicin and 91.4 ± 61.4 spikes (paired *t*-test *P*<0.01) during the first 20 s of the response to this agent. Where the receptors had ongoing activity, it was confirmed that the spontaneous NTIs occurring immediately before an electrical stimulus, or in the interval between the electrical stimulus and the expected time of occurrence of the electrically evoked NTI, occluded the electrically evoked NTI. This finding is consistent with collision between the orthodromic spontaneous NTI and antidromic electrically evoked NTI (see [13]). In all cases, both the ongoing and electrically evoked NTIs ceased after the period of excitation produced by capsaicin. This is likely to be due to strong depolarization of the nerve terminals by capsaicin that rendered them unexcitable [26].

#### Functionally defined corneal polymodal nociceptors express TRPV1 and have ramifying endings

**Fig 5:**
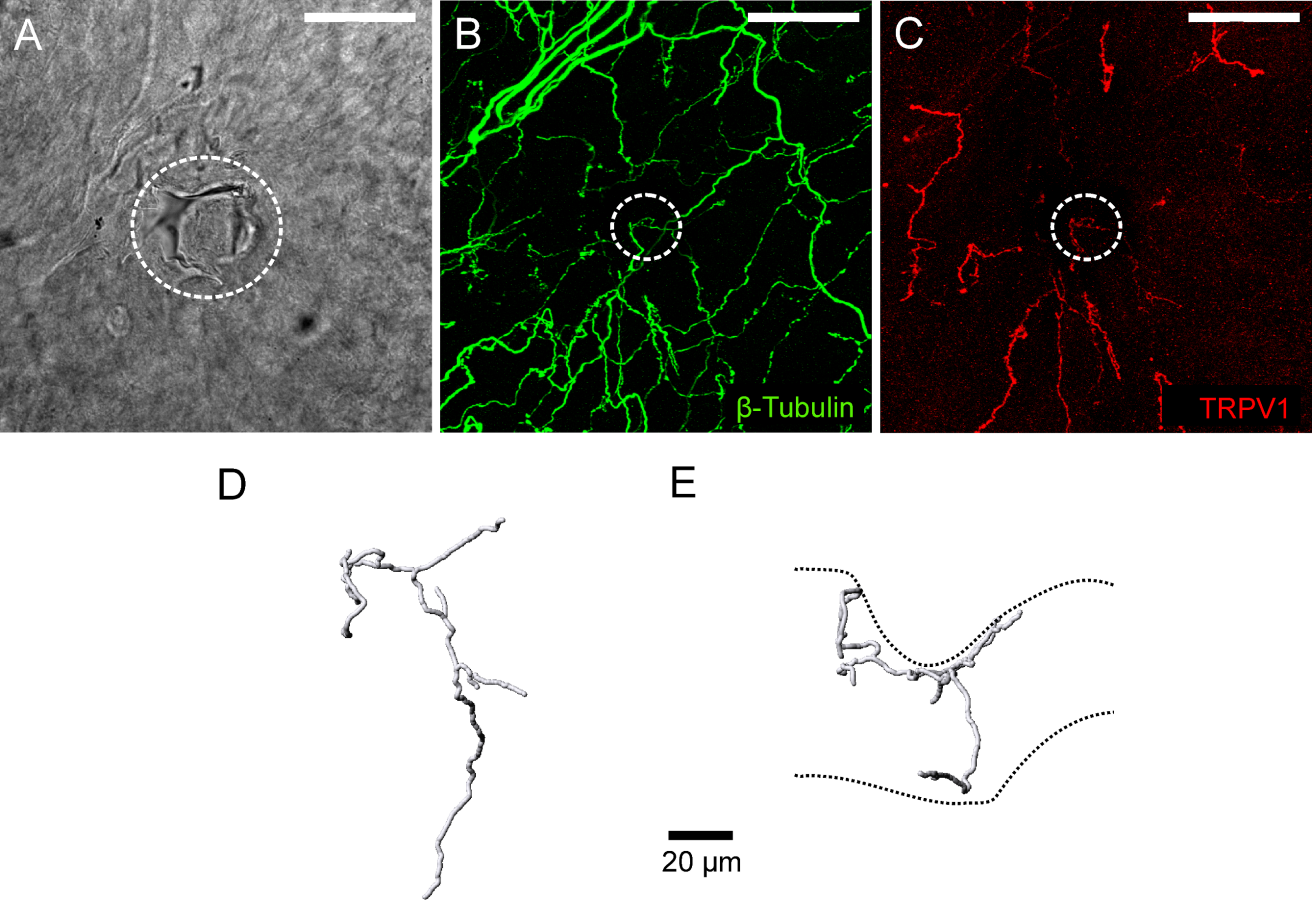
Immuno-labeling for TRPV1 at the polymodal receptor recording site where the electrical activity shown in Fig 4 was recorded. A, bright-field image showing within the white dotted circle the mark left on the corneal surface by the rim of the recording electrode. B, all axons in the field of view were revealed by β-tubulin III-IR (green). C, a TRPV1-IR (red) nerve terminal was located at the recording site. In B and C, the dotted circle approximates the dimensions of the electrode tip at the recording site revealed in A. The scale bar in A–C = 50 μm. D and E, show a 3-dimensional reconstruction of the TRPV1-IR nerve terminal from front (D) and side (E) views. The dashed lines in E approximate the boundaries of the corneal epithelium. The indentation of the epithelium was produced by the recording electrode.

Immuno-labeling for TRPV1 was assessed at 6 polymodal nociceptor recording sites. In all cases, there was a TRPV1-IR nerve terminal axon located close to the surface of the cornea immediately beneath the recording electrode (for the receptor identified in Fig 4 see Fig 5). These tissues were also labeled for β-tubulin III (Fig 5B) and there were no terminals that were only immunoreactive for this protein at the surface of the cornea beneath the recording electrode. In all cases, the axon arising from the SBP that gave rise to the TRPV1-IR axon under the electrode branched as it approached the most superficial layer of the corneal epithelium. In the squamous cell layer, the terminating branches ran in parallel to the corneal surface (i.e. the terminals had ramifying morphology; Fig 5D and 5E). Due to the relatively weak TRPV1-IR of the axons in the SBP it was only possible to follow the axon branch that gave rise to the ramifying endings under the electrode back to the point where they originated in the SBP.

#### Functionally defined corneal polymodal nociceptors were not TRPM8-IR

**Fig 6:**
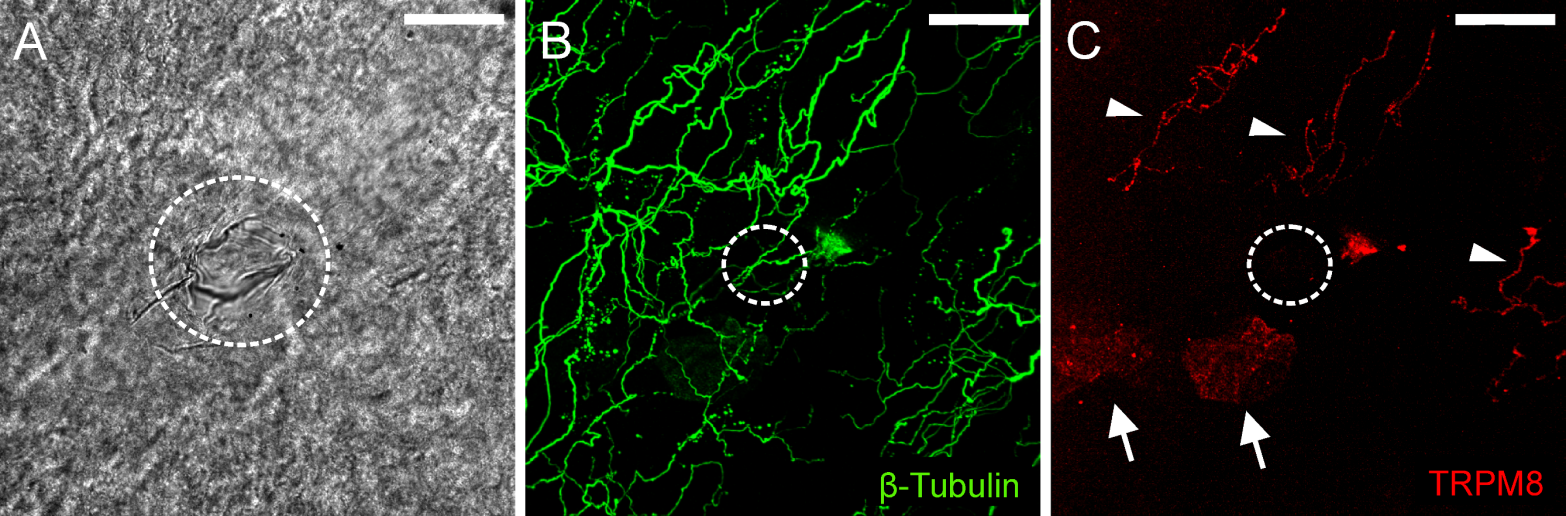
Immuno-labeling for TRPM8 at a polymodal receptor recording site. A, bright-field image showing within the white dotted circle the mark left on the corneal surface by the rim of the recording electrode. B, all axons in the field of view were revealed by β-tubulin III-IR (green). C, while TRPM8-IR axons (red) were located above and to the right of the recording site (indicated by arrowheads), those enclosed by the recording electrode were not immunoreactive for this protein. In C, there are small patches of fluorescence located on the surface of the cornea epithelium (arrows). These are an artifact produced by light reflecting off the surface of the epithelium. In B and C, the dotted circle approximates the dimensions of the electrode tip at the recording site revealed in A. The scale bar in A–C = 50 μm.

The remaining 3 polymodal nociceptor recording sites were assessed for TRPM8-IR. At these recording sites, there were β-tubulin III-IR axon terminals located close to the surface of the corneal epithelium immediately beneath the recording electrode, but none of these were TRPM8-IR (Fig 6). In all 3 cases, TRPM8-IR complex nerve endings were observed at sites a short distance from the recording site (Fig 6C).

## Discussion

The present study directly demonstrates for the first time that free nerve endings with specific functional properties in the cornea can be defined in accordance to their morphology and neurochemistry. Receptors with complex morphology and TRPM8-IR are cold thermoreceptors and those that have ramifying morphology and TRPV1-IR are polymodal nociceptors.

### Sensory nerve terminal morphology and neurochemistry

A previous study by Ivanusic et al. [17] demonstrated that TRPM8-IR corneal nerve endings in both the guinea pig and mouse corneal epithelium had almost exclusively complex morphology. The current study expands these findings and confirms that *functionally* identified cold thermoreceptors at the surface of the corneal epithelium have complex sensory nerve terminals that express TRPM8. None of the functionally identified cold thermoreceptors tested were TRPV1-IR. This is consistent with our earlier report that none of the TRPV1-IR terminals in the guinea pig or mouse corneal epithelium have complex morphology [16]. However, in guinea pig trigeminal ganglia, approximately 30% of the corneal sensory neurons that express mRNA for TRPM8 were TRPV1-IR [16]. These represent <10% of the total population of TRPV1-IR corneal sensory neurons. Because the antibodies used to identify TRPV1 and TRPM8 are raised in the same species we have not been able to identify nerve terminals that co-express both proteins in the corneal epithelium, but it has been suggested that they may be a subpopulation of polymodal receptors that are responsive to noxious cooling [27]. Furthermore, the small sample sizes used in the present study may not have been sufficient to allow identification of corneal afferent endings that co-express TRPV1 and TRPM8.

The nerve endings that had no or low levels of ongoing nerve activity, and that were excited by capsaicin, terminated in the squamous cell layer with ramifying morphology. These endings expressed TRPV1 but did not express TRPM8, and are most likely polymodal nociceptors (see Introduction). The morphology of these capsaicin-sensitive receptors is similar to that of the TRPV1-IR and glial cell line-derived neurotrophic factor receptor-α3 (GFRα3)-IR ramifying terminals identified in Alamri et al. [16]. None of the capsaicin-sensitive endings recorded from in the present study had the simple morphology of the TRPV1-IR, GFRα3-IR and CGRP-IR endings that terminate in the wing cell layer (see [16]). An attempt was made to determine if the functionally identified TRPV1-IR endings were also GFRα3-IR or CGRP-IR, but the immuno-labeling for these molecules did not survive the 3–4 hours that the corneas were maintained *in vitro* before fixation (i.e. in comparison with the findings reported in Alamri et al., 2015, little immuno-reactivity for GFRα3 or CGRP was observed in the corneal epithelium of the eyes studied *in vitro*). The selectivity of the recording electrode for the polymodal nociceptors with ramifying nerve endings in the squamous cell layer can be explained by the expected severe spatial attenuation of the extracellularly recorded electrical signals [28] arising from the endings of the simple TRPV1-IR nerve terminals in the wing and basal cell layers.

In addition to polymodal nociceptors and cold thermoreceptors, the corneal epithelium is supplied by sensory neurons that are only activated by mechanical stimuli (mechano-nociceptors) [11]. These neurons have thinly myelinated axons (Aδ-fibers) and the findings presented in Bron et al.[29] and Alamri et al. [16] demonstrate that many of the retrogradely labeled corneal sensory neurons that are NF200-IR (a marker for myelinated neurons) express mRNA for the mechano-sensor protein Piezo2. These Piezo2 expressing neurons do not co-express TRPV1 or TRPM8 [16,29] and for this reason it is hypothesized that they are the mechano-nociceptors. In preliminary experiments, commercially available antibodies to Piezo2 have been tested in the guinea pig and mouse cornea but no labeling of nerve terminals in the corneal epithelium was observed. In rabbits, MacIver and Tanelian [30,31] mapped the receptive site(s), on the surface of the corneal epithelium, of axons with Aδ-fiber conduction velocities that were activated by mechanical stimuli. At the receptive sites of these mechano-receptors they used the vital dye 4-di-ASP and epifluorescence microscopy to reveal that a single axon branched to form long (100–1200 μm) horizontal processes within the wing cell layer that run parallel to each other and to the corneal surface. Nerve terminals with this type of morphology have not been identified in either the guinea pig or mouse cornea using pan neuronal antibody markers [16, 17]. Using extracellular recording electrodes applied to the surface of the guinea pig corneal epithelium, sensory neurons that respond exclusively to mechanical stimuli are rarely recorded [13], which perhaps indicates that most of their sensory endings do not terminate superficially in the squamous cell layer.

### Functional significance of the different nerve terminal morphologies

The differing morphology of the nerve terminals formed by polymodal nociceptors and cold thermoreceptors identified in this study is presumably relevant to their function. For the ramifying nerve terminals of the polymodal nociceptors their location in the squamous cell layer suggests they sense changes at the surface of the cornea. However, as it was only possible to follow the axon branch giving rise to the ramifying endings under the electrode back to the point where they originated in the SBP, the full extent of the nerve terminals formed by the parent axon in the SBP has not been resolved. For this reason, it is not possible to speculate on how the ramifying morphology impacts on the integration of membrane potential changes induced by sensory stimuli or the initiation of action potentials.

In cold thermoreceptors, the location in the axon where the ongoing action potentials are initiated has been mapped and, in most cases, it is located at a point in the axon that is close to the recording site on the corneal surface [25]. Furthermore, the finding that the spontaneously occurring NTIs had smaller amplitudes than the electrically evoked NTIs indicates that they are initiated very close to the nerve terminal from which the recordings were made [25]. For the functionally identified cold thermoreceptors in the present study, there was a cluster of TRPM8 complex nerve terminal branches located in the immediate vicinity of the recording site. Each of these nerve terminal clusters was spatially isolated from other TRPM8-IR nerve terminal clusters in the epithelium. Furthermore, for 5 of the 6 functionally identified TRPM8-IR cold thermoreceptor endings, the cluster of nerve terminal branches could be identified as arising from a single parent axon in SBP that penetrated Bowman’s membrane close to the site of recording. Taken together, these findings suggest that each nerve terminal cluster behaves as a single sensory receptor.

The cluster of nerve terminal branches observed for the cold receptors in the cornea is likely to allow for efficient spatial summation of the receptor potential generated when multiple endings within the cluster are co-activated by sensory stimuli. Electrophysiological studies indicate that the membrane potential of cold thermoreceptor nerve endings is normally depolarized relative to that at the more proximal site in the axon where action potentials are initiated, and that this depolarization drives the ongoing nerve activity [14,15, 25]. Nerve terminals with high levels of ongoing activity characteristic of cold thermoreceptors are absent in the cornea of TRPM8 knockout mice [32]. This suggests that basal activity of the cold sensor ion channel TRPM8 is responsible for both the nerve terminal depolarization and the ongoing nerve activity. Furthermore, changes in the shape of NTIs indicate that warming hyperpolarizes the sensory nerve terminals of corneal cold thermoreceptors [15, 25], a finding that would be consistent with closure of TRPM8 channels. This suggestion is supported by the finding that blockade of TRPM8 with BCTC (N-(4-tertiarybutylphenyl)-4-(3-chloropyridin-2-yl) tetrahydropyrazine-1(2H)-carbox-amide–a TRPM8 antagonist) markedly reduced or silenced the ongoing activity of corneal cold thermoreceptors [32].

Recordings from the main peripherally projecting axon of cold thermoreceptors in the skin of cat’s nose indicate that they have a single punctate receptive field on the surface of the nose [3]. This study used serial section electron microscopy to demonstrate that below cold-receptive sites, an unmyelinated axon in the dermis divides into a cluster of short (50–80 μm) axonal branches that each terminate with large bulbar endings in the basal layer of the epithelium. These bulbar endings contained a great number of mitochondria and glycogen particles. The terminals of functionally identified cold thermoreceptors in cat sclera are also formed by axons that have swellings with accumulations of mitochondria, but the full 3-dimensional arrangement of these nerve terminals was not revealed [4]. The high density of mitochondria that has been observed in the nerve terminals of cold thermoreceptors may reflect the energy required to maintain the high levels of ongoing activity and to transduce sensory stimuli into action potentials (see [33]). In the present study, the nerve terminals of the corneal cold thermoreceptors had large *en passant* boutons and bulbar endings and this may be a structural specialization that allows a high density of mitochondria to be contained within the nerve terminals. Axon enlargement also greatly increases the surface area of the nerve terminal axons and potentially increases the number of sensory transducer molecules that can be expressed in the axolema per unit length of the axon.

As the receptors identified in this study are located superficially in the corneal epithelium they are likely to sense disease-related changes at the ocular surface. Indeed, current evidence indicates that cold thermoreceptors play a key role in regulating the basal secretion of tears and that changes in their function contribute to etiology of dry eye disease [12]. The possibility that changes to the morphology of the sensory nerve endings reported here contributes to ocular surface pathology needs to be investigated.

## Conclusion

In conclusion, it is often stated that the free nerve endings of C- and Aδ-neurons are ‘unspecialized’ but these findings in the cornea clearly indicate that at least some functionally defined receptor endings of these neurons have distinct nerve terminal morphologies. Present evidence indicates that the specialization of these nerve terminals is likely to relate to the function of the sensory receptor and their energy requirement.

## Supporting information

S1 Table Data for cold thermoreceptor nerve terminal impulses and observational findings for the nerve terminal located at each recording site. (PDF)

S2 Table Data for polymodal receptor nerve terminal impulses and observational findings for the nerve terminal located at each recording site. (PDF)
