# Supplementary material for: The neurochemistry and morphology of functionally identified corneal polymodal nociceptors and cold thermoreceptors
Source: PLoS One. 2018 Mar 28;13(3):e0195108. doi: 10.1371/journal.pone.0195108 (PMC5874071; doi:10.1371/journal.pone.0195108)
Supplement: S1 Table — (PDF) [file pone.0195108.s001.pdf]

S1 Table. Data for cold thermoreceptor nerve terminal impulses and observational findings for the nerve terminal located at each recording site.

	Latency of electrically evoked NTIs	Amplitude of spontaneous NTIs[#]	Amplitude of electrically evoked NTIs[#]	NTI frequency at basal temperature[†]	NTI frequency at the peak of the response to heating[†]	NTI frequency at the peak of the response to cooling[†]	Immuno-labelling primary antibodies*	Nerve terminal labelling at recording site	TRPM8 nerve terminal morphology[¶]
	**ms**	**µV**	**µV**	**Impulses/s**	**Impulses/s**	**Impulses/s**			
**C1**	8.9	57	98	10.8	0	14.8	TRPM8 & βTb	TRPM8 + βTb	Complex
**C2**	4.6	82	99	12	2.8	18	TRPM8 & βTb	TRPM8 + βTb	Complex
**C3**	10.4	94	114	8.6	0.8	11.8	TRPM8 & βTb	TRPM8 + βTb	Complex
**C4**	8.0	42	57	10	0	16	TRPM8 & βTb	TRPM8 + βTb	Complex
**C5**	9.7	66	90	5.2	0.2	11.2	TRPM8 & βTb	TRPM8 + βTb	Complex
**C6**	4.3	62	66	11	0	19.6	TRPM8 & βTb	TRPM8 + βTb	Complex
**C7**	6.6	54	59	4.6	0	19.2	TRPV1 & βTb	βTb	-
**C8**	8.1	30	53	10.4	2.4	14.2	TRPV1 & βTb	βTb	-
**C9**	8.0	25	47	7	0	11	TRPV1 & βTb	βTb	-
**C10**	7.8	26	47	8.4	0.2	16.8	TRPV1 & βTb	βTb	-
**C11**	7.1	37	51	9.4	0.2	14.6	TRPV1 & βTb	βTb	-
**C12**	12.2	68	79	14.4	0.4	18	TRPV1 & βTb	βTb	-
**Mean**	8.0	54	72	9.3	0.6	15.4			
**SD**	2.2	22	23	2.8	1.0	3.0			

NTI: Nerve terminal impulse

# The peak-to-peak amplitude of the diphasic spontaneous and electrically evoked NTIs were measured from averages of 25 NTIs. Before averaging the NTIs were aligned by their positive peak.

† The number of NTI during 5 seconds at the basal temperature (~32°C) and at the peak of the response to heating (to ~38°C) and cooling (to ~28°C) were measured.

* Primary antibodies: rabbit anti-TRPM8 (TRPM8), rabbit anti-TRPV1 (TRPV1), mouse anti-β-tubulin III (βTb). See Table 1 in paper for details of source and concentration.

¶ Complex nerve terminal morphology: the axon branch(es) from the subbasal plexus that gave rise to the TRPM8-immunoreactive axon terminals under the recording electrode had complex morphology with irregular large *en passant* boutons, short (< 5 µm) axonal side branches in the wing cell and squamous cell layers, and multiple bulbar endings located close to the surface of the corneal epithelium.
