# Supplementary material for: The neurochemistry and morphology of functionally identified corneal polymodal nociceptors and cold thermoreceptors
Source: PLoS One. 2018 Mar 28;13(3):e0195108. doi: 10.1371/journal.pone.0195108 (PMC5874071; doi:10.1371/journal.pone.0195108)
Supplement: S2 Table — (PDF) [file pone.0195108.s002.pdf]

S2 Table. Data for polymodal receptor nerve terminal impulses and observational findings for the nerve terminal located at each recording site.

	Latency of electrically evoked NTIs	Number of NTIs during 20 s before capsaicin	Number of NTIs during 20 s form start of capsaicin response#	Immuno-labelling primary antibodies*	Nerve terminal labelling at recording site	TRPV1 nerve terminal morphology¶
**P1**	14.5	14	161	TRPV1 & βTb	TRPV1+ βTb	Ramifying
**P2**	12.3	10	73	TRPV1 & βTb	TRPV1+ βTb	Ramifying
**P3**	8.8	6	166	TRPV1 & βTb	TRPV1+ βTb	Ramifying
**P4**	9.92	0	12	TRPV1 & βTb	TRPV1+ βTb	Ramifying
**P5**	7.8	0	180	TRPV1 & βTb	TRPV1+ βTb	Ramifying
**P6**	8.42	5	52	TRPV1 & βTb	TRPV1+ βTb	Ramifying
**P7**	13.3	2	81	TRPM8 & βTb	βTb	-
**P8**	9.6	0	53	TRPM8 & βTb	βTb	-
**P9**	17.7	0	45	TRPM8 & βTb	βTb	-
**Mean**	11.4	4.1	91.4			
**SD**	3.3	5.1	61.4			

NTI: Nerve terminal impulse

# The initiation of the response to capsaicin was defined by the point at which NTI frequency increased to >2 Hz.

* Primary antibodies: rabbit anti-TRPM8 (TRPM8), rabbit anti-TRPV1 (TRPV1), mouse anti-β-tubulin III (βTb). See Table 1 in paper for details of source and concentration.

¶ Ramify nerve terminal morphology: the axon arising from the subbasal plexus that gave rise to the TRPV1-immunoreactive axon at the recording site branched as it approached the most superficial layer of the corneal epithelium. In the squamous cell layer, the terminating branches ran in parallel to the corneal surface.
